# Spectral Karyotyping for identification of constitutional chromosomal abnormalities at a national reference laboratory

**DOI:** 10.1186/1755-8166-5-3

**Published:** 2012-01-16

**Authors:** Arturo Anguiano, Boris T Wang, Shirong R Wang, Fatih Z Boyar, Loretta W Mahon, Mohamed M El Naggar, Peter H Kohn, Mary H Haddadin, Vladimira Sulcova, Adam H Sbeiti, Mervat S Ayad, Beverly J White, Charles M Strom

**Affiliations:** 1 Quest Diagnostics Nichols Institute, San Juan Capistrano, CA 92690, USA

**Keywords:** Spectral Karyotyping, Marker Chromosome, FISH, array CGH

## Abstract

Spectral karyotyping is a diagnostic tool that allows visualization of chromosomes in different colors using the FISH technology and a spectral imaging system. To assess the value of spectral karyotyping analysis for identifying constitutional supernumerary marker chromosomes or derivative chromosomes at a national reference laboratory, we reviewed the results of 179 consecutive clinical samples (31 prenatal and 148 postnatal) submitted for spectral karyotyping. Over 90% of the cases were requested to identify either small supernumerary marker chromosomes (sSMCs) or chromosomal exchange material detected by G-banded chromosome analysis. We also reviewed clinical indications of those cases with marker chromosomes in which chromosomal origin was identified by spectral karyotyping. Our results showed that spectral karyotyping identified the chromosomal origin of marker chromosomes or the source of derivative chromosomal material in 158 (88%) of the 179 clinical cases; the identification rate was slightly higher for postnatal (89%) compared to prenatal (84%) cases. Cases in which the origin could not be identified had either a small marker chromosome present at a very low level of mosaicism (< 10%), or contained very little euchromatic material. Supplemental FISH analysis confirmed the spectral karyotyping results in all 158 cases. Clinical indications for prenatal cases were mainly for marker identification after amniocentesis. For postnatal cases, the primary indications were developmental delay and multiple congenital anomalies (MCA). The most frequently encountered markers were of chromosome 15 origin for satellited chromosomes, and chromosomes 2 and 16 for non-satellited chromosomes. We were able to obtain pertinent clinical information for 47% (41/88) of cases with an identified abnormal chromosome. We conclude that spectral karyotyping is sufficiently reliable for use and provides a valuable diagnostic tool for establishing the origin of supernumerary marker chromosomes or derivative chromosomal material that cannot be identified with standard cytogenetic techniques.

## Introduction

Spectral karyotyping is an invaluable diagnostic tool in constitutional studies for identifying marker chromosomes and chromosomal exchanges that are not fully defined by conventional cytogenetic methods [1,2]. This is especially true in cases involving *de novo *small supernumerary marker chromosomes (sSMCs) and derivative chromosomes [3-6]. Such definitive karyotyping is important in assessing risk for phenotypic abnormalities, especially for prenatal situations [7,8]. The ability to identify the origin of additional genetic materials is very important for providing information to couples in regard to the potential phenotypic and/or developmental effect of a *de novo *rearrangement. Similarly, in evaluation of infertility, the identification of derivative chromosomal material may shed light on the mechanism of infertility [9,10].

Although spectral karyotyping was developed more than a decade ago, few large-scale studies have assessed its ability to further resolve constitutional chromosomal rearrangements initially identified with conventional GTG-banding (G-banding) cytogenetic analysis. The primary aim of this study was to assess the utilization of spectral karyotyping for resolving chromosome abnormalities that are not well delineated by conventional G-banding.

## Materials and methods

### Spectral Karyotyping Analysis of Abnormalities not resolved with Conventional Chromosome Analysis

We reviewed the results of spectral karyotyping and confirmatory FISH testing performed on 179 consecutive clinical specimens (31 prenatal and 148 postnatal specimens) submitted to our national reference laboratory. In both prenatal and postnatal settings, the most common indication for spectral karyotyping analysis was the presence of chromosomal material not defined by conventional G-banding. Chromosomal abnormalities included unidentified marker chromosomes, additional rearranged material of unknown origin, ring chromosomes, and various complex rearrangements.

### Spectral Karyotyping Assay Procedure

The spectral karyotyping assay protocol recommended by the vendor (Applied Spectral Imaging, Carlsbad, CA) was followed. Emphasis was placed on the examination of telomeric regions. Spectral karyotyping was performed on metaphase chromosomes prepared for routine cytogenetic study from peripheral blood, amniotic fluid, and chorionic villus sampling(CVS) using standard hybridization procedures [1]. Equipment included the SKY Vision Cytogenetic Workstation with a SpectraCube^® ^and Sagnac interferometer, CCD camera for image capture, and a computer system for image analysis and pseudo-color karyotyping (Applied Spectral Imaging). The resulting multicolor images were examined with a 60x plan apochromatic objective followed by Kodak color print documentation of pseudo-colored karyotypes. All spectral karyotyping findings were confirmed by FISH using appropriate probes.

## Results

**Figure 1:**
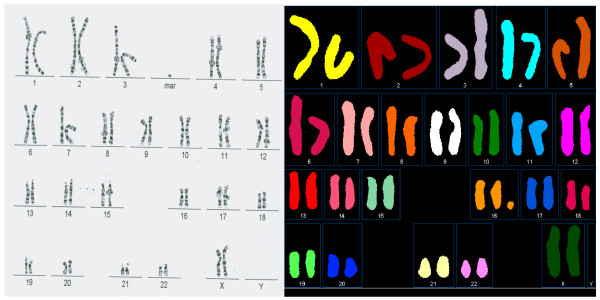
**A supernumerary ring marker originating from chromosome 16**. The sample tested was amniotic fluid. Banded metaphase (left) and spectral karyotype (right) are shown. Marker origin was confirmed by FISH using a chromosome 16 centromeric probe (not shown).

**Figure 2:**
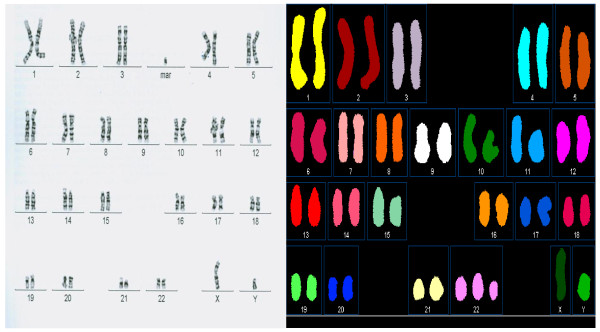
**A supernumerary bisatellited marker originating from chromosome 22 detected in a peripheral blood sample from a 2-year-old boy**. Banded metaphase (left) and spectral karyotype (right) are shown. Marker origin was confirmed by FISH using probes for 14/22 centromeres and the TUPLE1 gene locus (22q11.2) (not shown).

**Figure 3:**
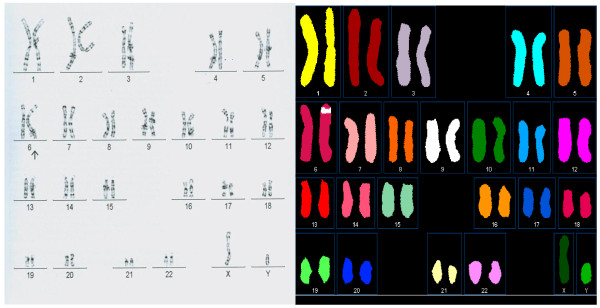
**An insertion of genetic material from chromosome 9 into chromosome 6**. The sample tested was peripheral blood from a 1-year-old boy. Banded metaphase (left) and spectral karyotype (right) are shown. The insertion was confirmed by FISH using painting probes specific for chromosomes 6 and 9 (not shown).

**Figure 4:**
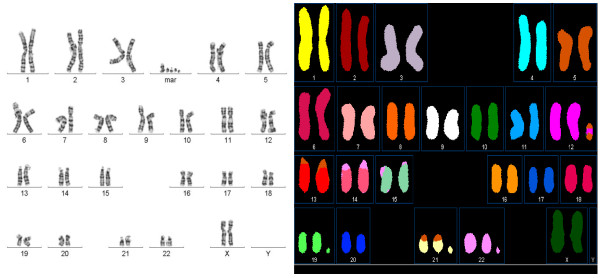
**Spectral karyotype analysis identified origins of multiple (3-6) markers present in a 3-year-old girl**. The sample was peripheral blood. Banded metaphase (left) and spectral karyotype (right) are shown. Spectral karyotype analysis indicated that the markers were respectively derived from chromosomes 12, 19, 21, 22, and X.

Most of the 179 clinical samples were submitted to further delineate additional material detected on G-banded analysis. Overall, spectral karyotyping identified the origins of the rearranged materials (including marker chromosomes) in 88% (158/179) of these cases; rates were similar in prenatal and postnatal cases (84% vs. 89%; see Table 1). The abnormalities included supernumerary marker chromosome (75 cases), additional material on a rearranged chromosome (71 cases), and ring chromosome (13 cases); a variety of complex rearrangements were observed in the remaining 20 cases. Spectral karyotyping identified the origin of the additional material in 77% to 100% of prenatal and postnatal samples, depending on the type of rearrangement involved. Notably, spectral karyotyping resolved the G-banding ambiguities in 19 of the 20 cases (95%) with complex rearrangements. Most cases in which the origin could not be identified were characterized by either a small marker chromosome present at a very low level of mosaicism (< 10%), or by a very small abnormality (data not shown). The lower practical limit of detection by spectral karyotyping appears to be within a single euchromatic band at a 500 band-level of resolution, or 6-10 Mb in size. Figures 1, 2, 3 and 4 illustrate the use of spectral karyotyping in a variety of selected cases with ambiguous GTG-banding results. Table 2 listed clinical indications of 41 cases referred for further characterization of sSMC and also other rearrangements by spectral karyotyping. Clinical indications for prenatal cases were mainly for marker identification after amniocentesis. For postnatal cases, the primary indications were developmental delay and multiple congenital anomalies (MCA). The most frequently encountered markers were of chromosome 15 origin for satellited chromosomes, and chromosomes 2 and 16 for non-satellited chromosomes. We were able to obtain pertinent clinical information for 47% (41/88) of cases with an identified abnormal chromosome.

**Table 1:** Spectral Karyotype Findings in 31 Prenatal Cases and 148 Postnatal Cases with Abnormal Karyotypes

Cytogenetic (GTG-banded) Abnormality	Spectral Karyotype Findings		
	
	Positive, n	Inconclusive, n	Approximate Sensitivity, %
Supernumerary Marker			
Prenatal (n = 13)	10	3	77
Postnatal (n = 62)	48	14	77
Additonal genetic material			
Prenatal (n = 6)	5	1	83
Postnatal (n = 65)	63	2	97
Ring chromosome			
Prenatal (n = 4)	4	0	100
Postnatal (n = 9)	9	0	100
			
Complex rearrangements			
Prenatal (n = 8)	7	1	88
Postnatal (n = 12)	12	0	100
========================	========	==========	=========
Total			
Prenatal (N = 31)	26	5	84
Postnatal (N = 148)	132	16	89

**Table 2:** Clinical indications of 41 cases referred for spectral karyotyping

Case number	Sex	Age	Type of Study	Result	Clinical indication
F0414116	M	Prenatal	Prenatal (AF)	der(1)	Supernumerary mosaic marker found elsewhere at amniocentesis, Parental chromosome studies reported as normal (studied elsewhere)

F0425680	F	Prenatal	Prenatal (CVS)	der(2)	Advanced maternal age

F0439968	M	1Y	Neonatal	r(2)	Developmental delay

F0533491	F	10Y	Postnatal	der(2)	Short stature, Pituitary dwarfism

F0549217	M	19M	Postnatal	der(2)	Delayed milestones, Multiple congenital anomalies

F0613262	M	2Y	Postnatal	der(3)	Developmental delay

F0732043	M	2Y	Postnatal	der(3)	Delayed milestones

F043725	M	2Y	Postnatal	der(7)	Prenatal ultrasound with an unspecified kidney abnormality; AF study with a result of 47,XY+mar[3]/46,XY[27]

F0451268	M	1Y	Neonatal	r(8)	47,XY,+r/46,XY (diagnosed elsewhere)

F0633932	M	2Y	Postnatal	der(9)	Developmental delay

F0713569	F	6M	Neonatal	i(9)(p10)	Macroglossia

F0716379	F	11Y	Postnatal	der(9)	Velocardiofacial (VCF) phenotype; NF1 diagnosis; Congenital cataract, Developmental delay, Abnormal aortic valve

F0646031	F	Prenatal	Prenatal (AF)	der(12)	Advanced maternal age

F0445519	M	2Y	Postnatal	der(14 or 22)	Developmental delay

F0519414	F	12Y	Postnatal	der(14 or 22)	Ataxia; Rule out 47,XXX

F058133	M	Prenatal	Prenatal (AF)	der(15)	Advanced maternal age; Amniocentesis study had a result of 47,XY,+mar[8]pat/46,XY[2]

F0554133	F	12Y	Postnatal	der(15)	Short stature

F0621647	F	12Y	Postnatal	r(15)	Previous study performed elsewhere

F0642559	F	3Y	Postnatal	psu idic(15)	Rule out SRY deletion

F0658799	M	6Y	Postnatal	idic(15)	Pervasive developmental disorder

F077829	M	25Y	Postnatal	der(15)	Infertility

F047204	F	33Y	Postnatal	r(16)	Multiple miscarriages

F0548089	F	2M	Neonatal	der(16)	Prenatal karyotype of 47,XX,+mar[7]/46,XX[12] (performed elsewhere)

F0616779	F	2Y	Postnatal	r(16)	Seizures

F0751841	F	Prenatal	Prenatal (AF)	der(16)	Advanced maternal age

F04635	F	7Y	Postnatal	der(18)	Previous blood study showed mosaicism for a small marker chromosome

F0528563	M	2M	Neonatal	der(18)	Multiple congenital anomalies; A ring chromosome detected at prenatal diagnosis

F077572	F	Prenatal	Prenatal (AF)	der(18)	Mosaic fetal karyotype of 47,XX,+mar[8]/46,XX[7] reported elsewhere; Advanced maternal age

F0534481	F	35D	Neonatal	der(19)	Amniotic fluid study had a karyotype of 47,XX,+mar

F0628560	F	2Y	Postnatal	r(19)	Multiple congenital anomalies

F0642312	F	12Y	Postnatal	der(19)	Rule out Fragile × syndrome

F0549359	N/G	10Y	Postnatal	der(20)	Developmental delay, Autism

F0819257	F	8Y	Postnatal	der(20)	Mental retardation/developmental delay, Trigonocephaly

F067630	F	11Y	Postnatal	r(21)	Dermatitis, Acquired acanthosis nigricans

F0535997	M	35Y	Postnatal	der(22)	Unspecified anterior pituitary hyperfunction, Chronic lymphocytosis, Thyroiditis, Hirsutism, Celiac disease, Disorders of iron metabolism.

F0560040	F	27Y	Postnatal	dic(22)	Bone marrow aspirate: agranulocytosis, neutropenia and borderline anemia; Blood specimen: agranulocytosis; To rule out a constitutional marker chromosome

F0636127	M	1Y	Postnatal	der(22)	Developmental delay

F0636122	M	N/G	Postnatal	der(22)	Rule out Trisomy 13

F0646601	F	11Y	Postnatal	idic(22)	Delayed milestones, Multiple congenital anomalies

F0845840	F	1D	Neonatal	der(22)	Microcephaly

F0851097	F	20Y	Postnatal	der(22)	Congenital heart defect

## Discussion

The findings of this study demonstrate the accuracy and usefulness of spectral karyotyping in identifying derivative constitutional chromosomal material. Our findings clearly demonstrate the clinical usefulness of spectral karyotyping for resolving the origin of a marker chromosome or rearranged chromosomal material when G-banding analysis does not. Once the source has been identified by spectral karyotyping, confirmatory testing using FISH probes or family studies should follow. In our series, these confirmatory studies verified informative spectral karyotyping results in all 158 cases. Therefore, if further data support the accuracy of spectral karyotyping, follow-up FISH testing may not always be needed for confirmation. In comparison with array CGH, spectral karyotyping is considered more valuable when it is used to identify polyploidy (e.g., 69,XXX), the origin of a marker chromosome in metaphase, or balanced rearrangements such as translocations, provided that the size of the subtle rearrangement is at least one-band size at a 500 band-level of resolution [9,11]. Spectral karyotyping may also be advantageous over array CGH when a low level of mosaicism (< 20%) is suspected, when a complex karyotype is observed on a conventional G-banded study, or when a DNA sample is not attainable for array CGH as a part of the follow-up process [12,13]. However, unlike array CGH, spectral karyotyping has limited ability to accurately measure the size of a chromosomal rearrangement including sSMCs and to precisely determine the chromosome regions or breakpoints involved [9]. As such, spectral karyotyping will not allow for an accurate correlation between a chromosomal rearrangement and a specific phenotype. It is through the effort made by Liehr et al. at Jena University of Germany since 2004 that a comprehensive sSMC homepage http://www.uniklinikum-jena.de/fish/sSMC.html has become available as an important reference source for the research community [14]. This homepage is a regularly updated, freely available online database with a focus to collect all available reported small supernumerary marker chromosomes (sSMCs) for future prognosis and as a helpful tool in genetic counseling.

In the 41 referral cases available for collection of further clinical information in our series, clinical indications were different between pre- and post-natal cases. While clinical indications were mainly for marker characterization after amniocentesis among prenatal cases, developmental delay and multiple congenital anomalies were the two major indications in our postnatal cases. Our data showed that the most frequently encountered markers were of chromosome 15 origin for satellited chromosomes, and chromosomes 2 and 16 for non-satellited chromosomes. These data are consistent with those published by Liehr et al [14]. Due to our unique setting as a national reference laboratory, we were only able to obtain pertinent clinical information for 47% (41/88) of cases with an identified abnormal chromosome.

Besides spectral karyotyping, there are many other approaches that are available for sSMC characterization. These approaches include centromere-specific multicolor FISH (cenM-FISH), subcentromere-specific multicolor (subcenM-FISH), microdissection and reverse FISH, microdissection and array CGH, or array CGH alone [14-18]. For other complex rearrangements, many possibilities are also accessible for further delineation after the chromosomal origin has been identified. These include BAC-FISH and FISH-banding methodologies such as multicolor banding fluorescence in situ hybridization (MCB/m-banding) up to array-CGH (microarray-based comparative genomic hybridization) in case of unbalanced rearrangements [16,18].

In recent years, array-CGH has attracted a great deal of research interest and is now considered an efficient and sensitive technique for detecting genome-wide copy number alterations at high resolution. It has increasingly replaced the role of spectral karyotyping in the diagnostic arena to identify the origin of sSMCs and other rearrangements, and also to characterize complex sSMCs. However, spectral karyotyping, to our belief, will still hold its unique value in the market place as a useful diagnostic tool, especially in identifying complex rearrangements in metaphase.

The data presented here and in the literature do not support the use of spectral karyotyping as a primary diagnostic tool in prenatal situations, as the risk of misdiagnosis is still a major concern [7,8]. Yaron et al. tested the application of spectral karyotyping for characterizing *de novo *small supernumerary marker chromosomes (sSMCs) during prenatal diagnosis [7]. In this series, the evaluation of ring chromosomes and non-satellited SMCs benefited greatly from the additional analysis; however, spectral karyotyping did not provide further information for characterization in two cases with bisatellited SMCs [7]. Besides, spectral karyotyping results, unlike array CGH, often need to be refined by the use of FISH probes. Therefore, Heng et al. proposed a strategy that combines G-banding, spectral karyotyping and FISH for pre- and post-natal chromosomal analysis [11]. This strategy involves routine G-banding for the initial evaluation, but any marker chromosomes or complex rearrangements thus detected are further characterized by spectral karyotyping and then verified with FISH. Our findings provide evidence that this strategy is efficient for pre- and postnatal analysis. In this regard, Liehr et al. (2009) also proposed a different approach that uses only FISH methologies to handle sSMC cases in prenatal situations [14].

In the foreseeable future, spectral karyotyping in conjunction with array CGH will continue to be applied not only in constitutional chromosome studies, but also in cancer cytogenetic studies for identification of complex and cryptic rearrangements [19]. Knowing that chromosomal changes in cancer are often considered a useful reference for therapy design, these chromosomal changes must be elucidated as precisely as possible. To provide better care for cancer patients, spectral karyotyping in combination with other molecular cytogenetic methodologies such as FISH and array CGH should be useful for supplementing G-banding analysis for the identification of significant prognostic rearrangements.

In conclusion, our findings describe the validation of spectral karyotyping in a national-wide commercial cytogenetics laboratory and its successful introduction into the diagnostic arena. This technology provides a valuable diagnostic tool for establishing the origin of small supernumerary marker chromosomes (sSMCs) and derivative chromosomal material that cannot be identified with standard techniques.

## Competing interests

The authors declare that they have no competing interests.

## Authors' contributions

AA supervised the assay implementation and its performance, supervised molecular cytogenetic studies, drafted and revised the manuscript. BTW reviewed and analyzed the spectral karyotyping data; drafted and finalized the manuscript. SRW supervised the molecular cytogenetic studies. FZB supervised the molecular cytogenetic studies and made critical comments on the drafted manuscript. LOM collected further clinical information and constructed Table 2. MME reviewed cases referred for spectral karyotyping analysis. PHK reviewed cases referred for spectral karyotyping analysis. MHH reviewed cases referred for spectral karyotyping analysis. VS carried out the molecular cytogenetic studies. AHS supervised the molecular cytogenetic studies. MSA supervised the molecular cytogenetic studies. BJW reviewed cases referred for spectral karyotyping analysis. CMS corresponding author; supervised the overall molecular cytogenetic studies and drafted the initial manuscript. All authors have read and approved the final manuscript.
